# Prediction of hospital visits for the general inpatient care using floating catchment area methods: a reconceptualization of spatial accessibility

**DOI:** 10.1186/s12942-020-00223-3

**Published:** 2020-07-27

**Authors:** J. Bauer, D. Klingelhöfer, W. Maier, L. Schwettmann, D. A. Groneberg

**Affiliations:** 1 Division of Health Services Research, Institute of Occupational Medicine, Social Medicine and Environmental Medicine, Goethe University, Theodor Stern Kai 7, 60590 Frankfurt, Germany; 2 Institute of Health Economics and Health Care Management, Helmholtz Zentrum München-German Research Center for Environmental Health (GmbH), Ingolstädter Landstr. 1, 85764 Neuherberg, Germany; 3 Department of Economics, Martin Luther University Halle-Wittenberg, 06099 Halle an der Saale, Germany

**Keywords:** Spatial accessibility, Floating catchment area, Hospital visits, Prediction, Need

## Abstract

**Background:**

The adequate allocation of inpatient care resources requires assumptions about the need for health care and how this need will be met. However, in current practice, these assumptions are often based on outdated methods (e.g. Hill-Burton Formula). This study evaluated floating catchment area (FCA) methods, which have been applied as measures of spatial accessibility, focusing on their ability to predict the need for health care in the inpatient sector in Germany.

**Methods:**

We tested three FCA methods (enhanced (E2SFCA), modified (M2SFCA) and integrated (iFCA)) for their accuracy in predicting hospital visits regarding six medical diagnoses (atrial flutter/fibrillation, heart failure, femoral fracture, gonarthrosis, stroke, and epilepsy) on national level in Germany. We further used the closest provider approach for benchmark purposes. The predicted visits were compared with the actual visits for all six diagnoses using a correlation analysis and a maximum error from the actual visits of ± 5%, ± 10% and ± 15%.

**Results:**

The analysis of 229 million distances between hospitals and population locations revealed a high and significant correlation of predicted with actual visits for all three FCA methods across all six diagnoses up to ρ = 0.79 (p < 0.001). Overall, all FCA methods showed a substantially higher correlation with actual hospital visits compared to the closest provider approach (up to ρ = 0.51; p < 0.001). Allowing a 5% error of the absolute values, the analysis revealed up to 13.4% correctly predicted hospital visits using the FCA methods (15% error: up to 32.5% correctly predicted hospital). Finally, the potential of the FCA methods could be revealed by using the actual hospital visits as the measure of hospital attractiveness, which returned very strong correlations with the actual hospital visits up to ρ = 0.99 (p < 0.001).

**Conclusion:**

We were able to demonstrate the impact of FCA measures regarding the prediction of hospital visits in non-emergency settings, and their superiority over commonly used methods (i.e. closest provider). However, hospital beds were inadequate as the measure of hospital attractiveness resulting in low accuracy of predicted hospital visits. More reliable measures must be integrated within the proposed methods. Still, this study strengthens the possibilities of FCA methods in health care planning beyond their original application in measuring spatial accessibility.

## Background

Adequate allocation of inpatient care resources requires assumptions about the need for health care and how this need will be met. Therefore, it is essential to correctly measure the need for health care. Otherwise, allocation of health care resources (such as hospital beds) will be based on false assumptions. Allocated health care resources must further be accessible to meet the need for health care. Access to inpatient care describes the process of patients in need meeting hospitals, qualified to provide the appropriate medical care. This complex process consists of a variety of social, financial, geographical, and personal factors [1]. There are five dimensions influencing access to health care providers: availability, accessibility, accommodation, affordability, and acceptability [2]. These dimensions reflect both spatial (availability and accessibility) and non-spatial factors (accommodation, affordability and acceptability). Accommodation accounts for the organization of health care (e.g. opening hours), affordability accounts for the financial aspects (e.g. health care insurance), and acceptability accounts for patient preferences. The non-spatial dimensions of access have already been shown to influence access to health care [3–5]. The spatial dimensions availability (i.e. number of health care providers) and accessibility (i.e. travel costs in terms of distance) are commonly combined and referred to as ‘spatial accessibility’ [6].

Measuring spatial accessibility can be done by calculating simple provider-to-population ratios, distances to the closest provider, or distances to the closest set of providers. Such measurements are commonly applied in health care research [7, 8]. More developed measures of spatial accessibility are based on gravity models [6, 9]. In contrast to simpler measures (e.g. population to provider ratios), gravity models account for distance decay and the delivery of care beyond administrative boundaries. This enables gravity models to return more realistic and accurate results [6, 9, 10].

The measurement of need for health care is more difficult. Traditionally, there are two theories describing the need for health care: the humanitarian and the realistic theory [11]. In a nutshell, the *humanitarian theory* defines the need for health care as any disturbance of the individuals‘wellbeing, regardless of the relief potential. In contrast, the *realistic theory* considers need for health care only if there is a potential to medical relief. For example, the inpatient care sector cannot relief the symptoms caused by the common cold in a young healthy individual. Therefore, this individual may have a need for inpatient care regarding the humanitarian theory, but not the realistic theory. Following this, a proper utilization of medical care is depending on the definition applied. In Germany as well as in other countries, the realistic theory is commonly used for the definition of the need for health care [9, 12, 13]. Consequently, the treatable morbidity—determined by current evidence-based knowledge—can be used as a surrogate marker for this need.

Common concepts of hospital planning often consider outdated methods to measure health care need and spatial accessibility. For example, hospital planning in Germany is commonly based on the Hill-Burton formula [14]. This formula takes the number of cases, population size, length of stay and use of hospital beds into account [15]. The need for health care is often operationalized by simple population counts per administrative area. Thus, omitting the treatable morbidity as the more appropriate proxy. Furthermore, the closest provider approach is often used as the measure of spatial accessibility, which projects all modelled demand on the closest provider [7, 9, 16]. Following the realistic theory, these approaches do not adequately reflect the concept of need for health care and spatial accessibility. Therefore, it is questionable if current concepts of hospital planning allow an adequate allocation of hospital resources [14]. Floating Catchment Area (FCA) methods (as a special case of a gravity model) could improve this allocation process since they integrate both the concept of the need for health care (as suggested by the realistic theory) and a sophisticated measure of spatial accessibility [17–19]. Delamater et al. further showed that FCA methods can provide predictions of patient utilization patterns [20]. Using FCA methods to predict hospital visits could be essential for the planning of future health care resource allocation. Examples for FCA methods are the ‘integrated floating catchment area’ (iFCA) methodology, the ‘Modified 2 Step Floating Catchment Area’ (M2SFCA) methodology, or the ‘Enhanced 2 Step Floating Catchment Area’ (E2SFCA) methodology [10, 21, 22]. Each of which has its merits: The iFCA method uses a variable distance decay function, the M2SFCA method accounts for the suboptimal distribution of health care resources, and the E2SFCA method represents an earlier member of the FCA family that has been widely used in health care research [10, 21, 22].

In Germany, the application of FCA methods to predict the need for health care has not been studied so far. In general, the German health care system is funded by a statutory contribution system, divided between statutory and private health insurance funds. These compulsory health insurance funds ensure the affordability of health care in Germany, which had the highest healthcare expenditure relative to gross domestic product (GDP) in 2017 (11.3%) among all member countries of the European Union [23]. Regarding the inpatient sector, health care in Germany is provided by both public and private hospitals. Due to the immense financial dimension of hospital care, it is crucial to adequately allocate resources. Hence we evaluated the feasibility and the potential performance of FCA methods to predict the need for health care for the inpatient sector in Germany.

## Methods

The analysis was performed for medical specialties providing the mainstay of inpatient care in Germany: surgery and internal medicine. In addition, we included neurology as a minor specialty. The study area (national boundaries of Germany) was subdivided into a one-by-one kilometer grid. We excluded all grid cells not accessible by car (e.g. cells covering water bodies or forests). Grid cells were used instead of predefined boundaries since grid cells all have the same size allowing for easy comparison. Furthermore, they are stable over time [24].

### Data

For each specialty (surgery, internal medicine, and neurology), we retrieved the locations (addresses) of the hospitals as of 2017 and the number of hospital beds. Data were taken from the hospital directory of the German Federal Statistical Office [25]. We further retrieved the two most frequent diagnoses based on the ICD10-Code as stated by the Federal Statistical Office for 2017 for each specialty: For internal medicine, this corresponded to heart failure (I50: n = 464,724) and atrial flutter/fibrillation (I48: n = 313,462), for surgery to femoral fracture (S72: n = 188,490) and gonarthrosis (M17: n = 186,773), and for neurology to stroke (I63: n = 259,594) and epilepsy (G40: n = 147,685). Hospitals were excluded if no respective main diagnosis was coded in 2017. We retrieved the number of ICD10-counts on district level from the Diagnosis-Related Groups Statistic provided by the Federal Statistical Office [26]. These ICD10-counts were used as the proxy for the need for health care. The ICD10-counts were provided both by 5-year age groups (1-4 years, 5-9 years, 10-14 years etc.) and by sex (male or female) on national level. In order to enhance resolution, we used the respective ICD10-counts on district level to proportionally disaggregate the data on municipality level by both age and sex. From there we evenly assigned each km^2^-grid cell the ICD10-count based on the number of grid cells within a municipality. Such disaggregation methods are commonly applied in the absence of geocoded microdata [24]. In order to normalize the ICD10-counts, we further calculated the ratio of ICD10-counts per km^2^ to the population size per km^2^. Hereby geographic variations of the disease burden will be revealed.

### Calculation of predicted hospital visits

The calculation of predicted hospital visits was based on the methodology of the floating catchment area (FCA) family metrics. These FCA methods represent a special case of a gravity model for the measurement of spatial accessibility. In simplified terms, all FCA methods compute a ratio of need for health care and health care capacity for each population location. We included the following FCA methods: iFCA, M2SFCA, and E2SFCA. For all FCA methods the predicted hospital visits *V* at hospital location *y* can be conceptualized by the summed need for health care from all population locations *x* (centroids of the km^2^-grid) that place their need for health care in terms of ICD10-counts (*P*_*x*_) on hospital *y* accounted for a weight factor *W* if this hospital is within catchment area (*C*).

1 $$V_{y} = \mathop \sum \limits_{{x \in \left( {d_{xy} \le C_{x} } \right) }} W \cdot P_{x}$$

The weight factor *W* represents the probability that the need for health care is placed on hospital *y* and differs among FCA methods (*W*_*iFCA*_, *W*_*M2SFCA*_, and *W*_*E2SFCA*_). The maximum catchment area (C_x_) was set to 120 min and covers the region with a maximum 120-min driving time between the hospital and population location by car on a road network with its specific speed limits. This catchment is in line with similar studies [27]. The actual driving time between hospital and population locations in minutes is represented by *d*_*xy*_. Regarding the capacity, we used the number of hospital beds for internal medicine, trauma surgery/orthopedics, and neurology. Hospitals without capacity regarding these specialties were excluded. Furthermore, we only included hospitals that were relevant for the delivery of care regarding the diagnoses. Therefore, hospitals with < 10 reported ICD10-counts in 2017 were excluded. These data were retrieved from the Quality Reports published by the German Federal Joint Committee as of 2017 [28]. Hospital beds have been shown to be accurate surrogates of facility size (capacity) and are commonly used in health care research and planning [29]. In the context of this study, hospital beds were used as a measure of hospital attractiveness: the more hospital beds were provided, the more attractive the hospital was for patients (i.e. higher probability of utilization) [30].

### iFCA

Distance decay is represented by a sigmoid function *f*_*iFCA*_ depending on the distance *d*. The decay function is based on the cumulative logistic distribution function. According to the gravity model, it represents the declining utilization rate of health care with increasing distance. The weight factor in this model depends on the distance between the population location, the hospital, and the number of hospital beds *S*_*y*_ (attractiveness of hospital *y*). The second part of formula 2 represents the Huff-Model and takes competition into account [31, 32]. It calculates the utilization probability of hospital *y* if there are competitive hospitals *z* within the catchment area of population location *x*. For the iFCA method, the corresponding weight factor is calculated as follow:

2 $$W_{iFCA} = f_{iFCA} (d_{xy} ) \cdot \left( {\frac{{S_{y} \cdot f_{iFCA} \left( {d_{xy} } \right)}}{{\mathop \sum \nolimits_{{z \in \left( {d_{xz} \le C_{x} } \right)}} S_{z} \cdot f_{iFCA} \left( {d_{xy} } \right)}}} \right)$$

### M2SFCA and E2SFCA

The M2SFCA and E2SFCA methods are described in detail elsewhere [10]. For the purpose of this study, we follow Delamater et al., who described the application of both M2SFCA and E2SFCA method to predict hospital visits [20]. To calculate predicted hospital visits *V* at hospital location *y* using the M2SFCA and E2SFCA methods, the accessibility indices (AI) have to be calculated first. The formula for calculating the *AI* is as follows:

3 $$AI_{M2SFCA} = \mathop \sum \limits_{{y \in \left( {d_{xy} \le C_{x} } \right) }} \frac{{S_{y} \cdot f_{M2SFCA} \left( {d_{xy} } \right)\cdot f_{M2SFCA} \left( {d_{xy} } \right)}}{{\mathop \sum \nolimits_{{x \in \left( {d_{xy} \le C_{x} } \right)}} P_{x} \cdot f_{M2SFCA} \left( {d_{xy} } \right)}}$$

4 $$AI_{E2SFCA} = \mathop \sum \limits_{{y \in \left( {d_{xy} \le C_{x} } \right) }} \frac{{S_{y} \cdot f_{E2SFCA} \left( {d_{xy} } \right)}}{{\mathop \sum \nolimits_{{x \in \left( {d_{xy} \le C_{x} } \right)}} P_{x} \cdot f_{E2SFCA} \left( {d_{xy} } \right)}}$$

The data inputs for *S*_*y*_, *P*_*x*_ and *C*_*x*_ were identical to those for the iFCA method. For the distance decay function *f*_*M2SFCA*_ in (3), we used a downward log logistic distance decay function with *a *= 13.39 and *ß *= 1.89. We used this parameter setting since it has been shown to provide the most predictive power compared to other settings [20]. For the distance decay function *f*_*E2SCFA*_ in (4), we used the Gaussian function with fast decay and four travel time zones since this decay function has been widely used with the E2SFCA method [20, 33, 34]. After computing the *AI* for each population location *x*, the partial *AI* has to be calculated.

5 $$partial \;AI_{M2SFCA} = \frac{{S_{y} \cdot f_{M2SFCA} \left( {d_{xy} } \right)}}{{\mathop \sum \nolimits_{{x \in \left( {d_{xy} \le C_{x} } \right)}} P_{x} \cdot f_{M2SFCA} \left( {d_{xy} } \right)}}$$

6 $$partial \;AI_{E2SFCA} = \frac{{S_{y} }}{{\mathop \sum \nolimits_{{x \in \left( {d_{xy} \le C_{x} } \right)}} P_{x} \cdot f_{E2SFCA} \left( {d_{xy} } \right)}}$$

The partial *AIs* in (5) and (6) represent the disaggregated accessibility indices before they are summed up to build the final *AI*. The weight factor can be described as the ratio of the partial and the final *AI*.

7 $$W_{M2SFCA} = \frac{{partial \;AI_{M2SFCA} }}{{AI_{M2SFCA} }}$$

8 $$W_{E2SFCA} = \frac{{partial\; AI_{E2SFCA} }}{{AI_{E2SFCA} }}$$

The major difference between the FCA methods is the distance decay function used and the integration of the Huff model within the iFCA method. For benchmark reasons, we additionally calculated the predicted hospital visits using the closest provider approach: we assigned the need for health care at population location *x* to its closest hospital *y*. This approach is regularly used within health services research as a simple and easily calculated measure of spatial accessibility. The predicted number of hospital visits was then compared with the actual number of hospital visits [20]. However, due to the exclusion criteria of hospitals described above, the sum of both numbers (predicted and actual visits) had to differ. Therefore, we factorized the predicted visits to equal the sum of both numbers.

To demonstrate the potential performance and feasibility of all methods mentioned above, we further predicted hospital visits by using the actual hospital visits as the measure of attractiveness *S*_*y*_. The actual hospital visits represent the best measure of hospital attractiveness since they are the result of all influencing factors, both spatial and non-spatial. In contrast to hospital beds, hospital visits account for multiple visits per year as well the bed utilization ratio. This approach is purely hypothetical since it requires the actual hospital visits to predict hospital visits. Therefore, it would be of no use for future predictions. However, it provides important information about the methods’ potentials if an adequate measure of hospital attractiveness is applied.

### Data analysis

Our primary outcome was the accuracy to correctly predict hospital visits. First, we performed a correlation analysis. Second, we performed a linear regression analysis with the dependent variable being the actual hospital visit count and the independent variable being the predicted hospital visit count. For the regression analysis, we visually tested homoscedasticity using a residuals plot. We further visually tested normality of the residues using a histogram of the standardized residues. Third, we defined correctly predicted visits as a maximum error from the actual visits of ± 5%, ± 10% and ± 15%, respectively, and calculated the ratio of correctly predicted visits to actual visits at hospital level.

We used ArcGIS Pro 2.4. (ESRI Inc., Redlands, USA) for the geospatial analysis and SPSS version 23 (IBM, Armonk, USA) for the correlation analysis. We calculated Spearman’s ρ for nonparametric data. We further applied the Kruskal–Wallis-Test to test for significant differences.

## Results

**Fig. 1:**
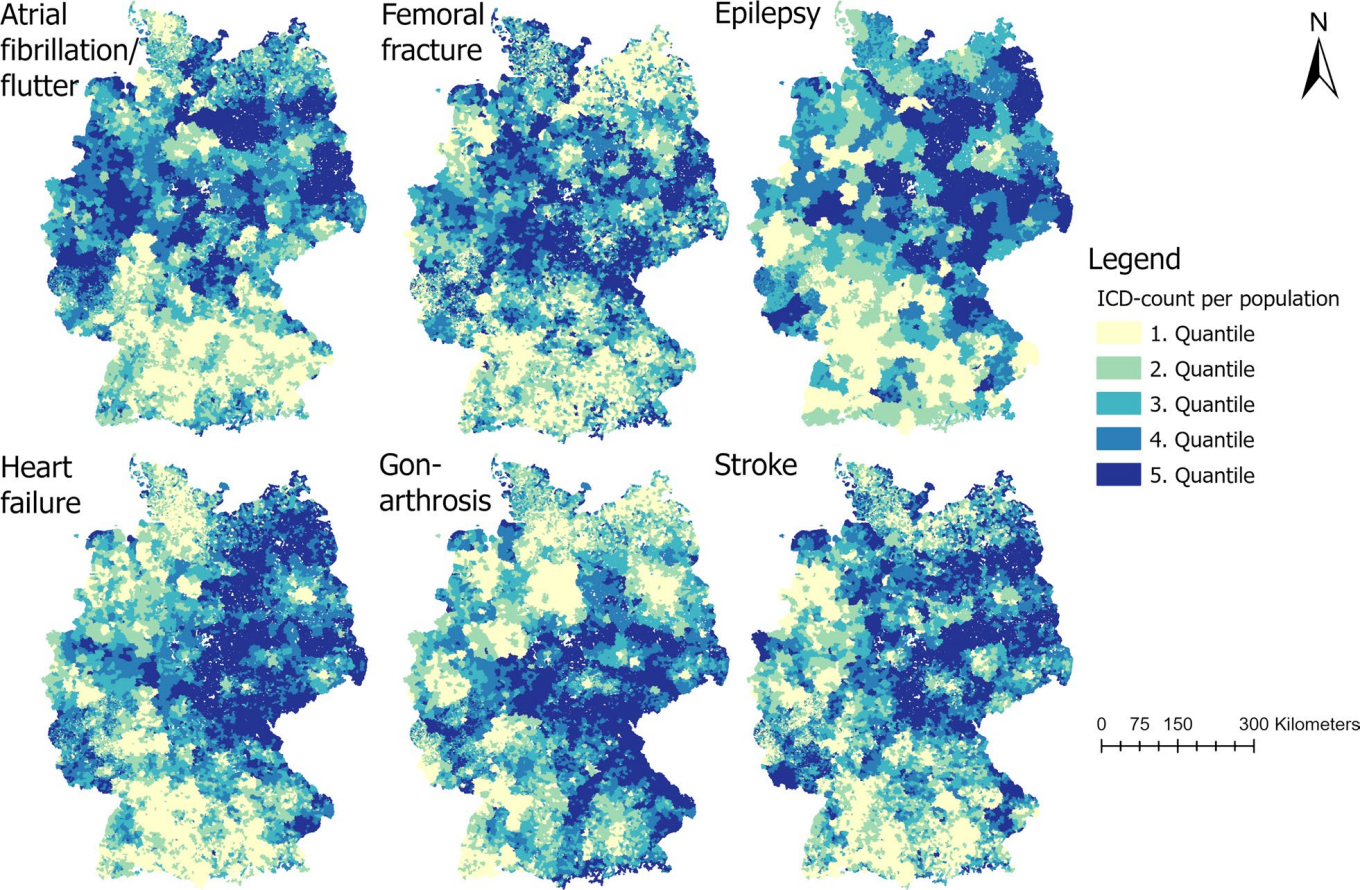
ICD-counts per population per km^2^

The normalized ICD10-counts per km^2^ are shown in Fig. 1. The visual analysis revealed varying geographic patterns for each diagnosis. For example, atrial flutter/fibrillation (I48) showed a clear north–south gradient with more ICD10-counts in the north of Germany. On the other hand, there was a clear east–west gradient for heart failure (I50) with higher ICD10-counts in the east.

The analysis utilized 229 million distances between hospital and population locations. The mean effective catchment area (i.e. where the decay function results in a factor less than 0.01) was 60 min. The resulting predicted hospital visits were highly correlated with the actual hospital visits as shown in Table 1. The correlation analysis showed a high and significant correlation for all three FCA methods across all six diagnoses up to ρ = 0.79 (p < 0.001). The M2SFCA method showed the highest correlations for all diagnoses except for femoral fracture (S72) and gonarthrosis (M17). However, all three FCA methods produced similar high correlation values, which suggested that all three FCA methods were similar regarding the prediction of hospital visits. This finding was supported by the statistical analysis which showed no statistically significant difference between the FCA methods regarding the predicted hospital visits. This applied to all six diagnoses.

**Table 1:** Correlation of predicted and actual hospital visits using hospital beds visits as measure of hospital attractiveness

Method	Heart failure	Atrial flutter/fibrillation	Femoral fracture	Gonarthrosis	Stroke	Epilepsy
iFCA	0.73*	0.68*	0.42*	0.34*	0.59*	0.59*
M2SFCA	0.79*	0.70*	0.42*	0.35*	0.63*	0.59*
E2SFCA	0.78*	0.69*	0.32*	0.36*	0.52*	0.52*
Closest Provider	0.48*	0.33*	0.51*	0.05	0.55*	0.36*

*iFCA* integrated floating catchment area, *M2SFCA* modified 2 Step floating catchment area, *E2SFCA* enhanced 2 step floating catchment area

* p < 0.001

Overall, FCA methods showed substantially stronger correlations with actual hospital visits compared to the closest hospital method (correlation up to ρ = 0.51 for femoral fracture (S72); p < 0.001).

The regression analysis (Table 2) revealed that for I50 using the M2SFCA method, 54.1% of the variance of hospital visits could be explained. However, for S72, only 9.2% of the variance of hospital visits could be explained using the E2SFCA method. Across all six diagnosis, the M2SFCA method returned the highest percentage of explained variance with a mean of 28,9% followed by the iFCA with 27.2% and the E2SFCA with 23.6%.

**Table 2:** Results of the regression analysis (r-squared values) of predicted and actual hospital visits using hospital beds visits as measure of hospital attractiveness

Method	Heart failure	Atrial flutter/fibrillation	Femoral fracture	Gonarthrosis	Stroke	Epilepsy
iFCA	0.524*	0.311*	0.185*	0.163*	0.140*	0.092*
M2SFCA	0.541*	0.330*	0.239*	0.335*	0.132*	0.155*
E2SFCA	0.473*	0.322*	0.238*	0.316*	0.132*	0.149*
Closest Provider	0.150*	0.027*	0.151*	0.002	0.256*	0.015**

*iFCA* integrated floating catchment area, *M2SFCA* modified 2 step floating catchment area, *E2SFCA* enhanced 2 step floating catchment area

* p < 0.001; ** p < 0.05

In Table 3, the results are shown using a 5%, 10% and 15% error. The analysis revealed that by using a 5% error, 4.3–13.4% of predicted visits were predicted correctly across all three FCA methods. The proportion of correctly predicted visits increased up to 32.5% for the M2SFCA method allowing a 15% error. Within this range of correctly predicted visits, the M2SFCA method performed the best. For femoral fracture (S72), however, using the closest provider approach returned the highest proportion of correctly predicted hospital visits compared to the FCA methods. This finding was in line with the results of the correlation analysis.

**Table 3:** Proportion of correctly predicted hospital visits according to the allowed error (5–15%) by diagnosis

	Heart failure	Atrial flutter/fibrillation	Femoral fracture	Gonarthrosis	Stroke	Epilepsy
5% error						
iFCA	8.7	6.9	6.6	4.5	7.0	9.4
M2SFCA	10.3	8.5	6.3	4.6	8.8	13.4
E2SFCA	10.8	6.1	5.5	5.6	8.5	8.8
Closest Hospital	8.3	4.3	9.9	3.6	8.1	8.0
10% error						
iFCA	16.3	13.4	12.5	9.2	17.1	21.4
M2SFCA	21.7	15.5	12.3	11.0	19.4	21.7
E2SFCA	19.6	13.0	10.7	10.2	14.9	17.3
Closest Hospital	15.5	10.1	18.4	7.1	16.5	16.3
15% error						
iFCA	24.6	21.0	19.4	14.8	28.4	27.1
M2SFCA	32.5	22.5	20.4	16.1	30.2	30.5
E2SFCA	30.0	18.5	16.2	15.0	24.2	25.1
Closest hospital	21.2	14.3	26.6	11.3	25.6	23.0

*iFCA* integrated floating catchment area, *M2SFCA* modified 2 step floating catchment area, *E2SFCA* enhanced 2 step floating catchment area

Regarding the overall absolute magnitude of the deviation: the highest positive deviation was present for I48 with 5255 hospital visits using the iFCA method. The highest negative deviation was present for I50 with 2666 using the iFCA method. However, it has to be mentioned that the magnitude of the deviation was similar across all three FCA methods.

We finally examined the potential performance of all methods by using the actual hospital visits as the measure of hospital attractiveness (Table 4). This analysis revealed a very strong correlation of predicted and actual hospital visits across all six diagnoses and all three FCA methods with up to ρ = 0.99 (p < 0.001). Since the actual hospital visits represent the result of the hospitals attractiveness, this hypothetical analysis demonstrated the potential benefit of the FCA method to predict hospital visits if more adequate measures of hospital attractiveness would be applied. Since the closest provider method does not include a distinct measure of hospital attractiveness, the results are identical to the correlation analysis shown in Table 1. This being said, the FCA methods performed particularly well compared with the closest provider method.

**Table 4:** Correlation of predicted and actual hospital visits using actual hospital visits as measure of hospital attractiveness within the FCA methods

	Heart failure	Atrial flutter/fibrillation	Femoral fracture	Gonarthrosis	Stroke	Epilepsy
iFCA	0.93*	0.97*	0.93*	0.94*	0.91*	0.91*
M2SFCA	0.97*	0.98*	0.95*	0.96*	0.93*	0.93*
E2SFCA	0.98*	0.99*	0.97*	0.98*	0.96*	0.96*
Closest Provider	0.48*	0.33*	0.51*	0.05	0.55*	0.36*

*iFCA* integrated floating catchment area, *M2SFCA* modified 2 step floating catchment area, *E2SFCA* enhanced 2 step floating catchment area

* p < 0.001

## Discussion

In this study, we were able to demonstrate the impact of FCA methods regarding the prediction of hospital visits by using hospital beds as the measure of hospital attractiveness. All examined FCA methods resulted in a high and significant correlation of predicted and actual hospital visits. However, the differences between the FCA measures were not statistically significant. Furthermore, our results suggested that FCA methods perform particularly well in non-emergency settings. As for emergency settings, the closest hospital method was a more appropriate measure. Finally, the accuracy of all measures examined seemed to be low using hospital beds as the measure of hospital attractiveness. Therefore, FCA methods as applied in this study are not suited to be applied in current health care planning. In order to draw a final conclusion regarding the utility of FCA methods in this context, further studies are needed. Still, the results of this study strengthen the potential of FCA methods in health care planning beyond their original application. As for the outpatient sector in Germany, FCA methods already are discussed to be the future of need-based regulations [9]. One major advantage of using FCA methods to predict hospital visits is their ability to account for spatial changes with high resolution (both at hospital and population level). This is especially important since there is an emerging trend to consolidate hospitals in developed countries in order to use synergetic effects [35]. Using FCA methods, the effects of changes regarding the number and distribution of hospitals as well as changes regarding the disease burden on the number of hospital visits can be measured. Based on such measurements, allocation of hospital resources could be more adequate and therefore, the related costs could be minimized. This is especially important for Germany with the highest healthcare expenditure relative to GDP in 2017 among all member countries of the European Union [23]. However, based on our results, the prerequisite for FCA measures to be integrated in current hospital planning is to find a more adequate measure of hospital attractiveness.

For femoral fracture as an emergency setting, the closest hospital method was the more appropriate measure to predict hospital visits since it performed better than all three FCA methods. For stroke, the closest provider method also performed particularly well. This could be due to the emergency nature of both diagnoses. In emergency settings, the choice of hospitals is based on different priorities compared to non-emergency settings: Spatial factors are commonly prioritized, whereas social or personal factors are omitted. This is mostly due to the time (and therefore distance) dependent mortality of many emergency settings like out of hospital cardiac arrest or polytrauma [36]. Since the other diagnoses often do not represent medical emergencies, minimizing travel time may not be the priority. As supported by the finding that the closest hospital method returned the highest correlation for femoral fractures, the closest provider approach represents the more accurate measure to predict hospital visits in emergency settings. In other words, our results suggest that FCA methods are best applied in non-emergency settings.

In our study, we used the number of hospital beds as the measure of attractiveness since it has been shown that hospital beds are an accurate and commonly used surrogate for hospital capacity and directly influence patient satisfaction [15, 20, 29, 37]. As shown in a recent study, the number of hospital beds (i.e. facility size) also directly impacts the patient choice for the first hospital visit [29]. However, it has to be noted that the number of hospital beds are also related to population size. This is for example reflected in current hospital planning in Germany within the Hill-Burton formula, which accounts for population size [14, 15]. Using hospital beds resulted in low accuracy of FCA methods to predict hospital visits. This may be because hospital beds are not the only influencing factor regarding the hospital choice and therefore hospital attractiveness. Studies have shown that the main non-spatial criteria for patients’ choice of a certain hospital were personal experience, recommendations, equipment and reputation of the hospital [4, 29, 30]. Due to these factors’ hospitals are not equally likely to serve the patients’ medical need. For example, hospitals specialized in gonarthrosis or atrial fibrillation treatment are more likely to be chosen by patients with the respective condition. This represents the ‘reputation of the hospital’ as mentioned above. Other studies found that the complication rate of a hospital as well as the readmission of patients represent major influencing factors [38, 39]. Ibrahim et al. examined the readmission rate of heart failure patients but failed to demonstrate an influence of medical scores on the 30-day readmission rate [39]. Still, medical aspects are likely to have an influence on the hospital choice and should therefore be considered whenever possible. This also applies for femoral fractures if the fracture is considered a major trauma requiring care in a major trauma center which would lead to the bypassing of a local hospital. For stroke, this translates in the ‘mothership model’ vs. the ‘drip and ship model’ [40]. This being said, spatial accessibility and therefore the approach applied in this study does not account for these medical aspects. We did not include further criteria for hospital attractiveness due to lack of data. However, using the actual hospital visits as the measure of attractiveness to predict the hospital visits enabled us to demonstrate the potential of the FCA methods. Actual hospital visits are the result of all influencing factors that eventually let patients choose a certain hospital. Therefore, it is reasonable to conclude that it was rather the parameter choice that led to low accuracy than the method itself. Future research should evaluate a combination of hospital attributes regarding its power to estimate hospital attractiveness. This estimate should also account for data availability, which may hinder its utilization. Integrating such an estimate within FCA methods will potentially result in more reliable predictions of hospital visits.

### Strengths and limitations

The FCA methods used in our study were already applied in a variety of settings including the inpatient as well as the outpatient sector [10, 41, 42]. In a similar study, it was shown that the M2SFCA method was able to correctly predict 74% of hospital visits using crude population counts in Michigan (USA) [20]. In our study, the proportion of correctly predicted hospital visits was considerably lower. However, first, the definition of correctly predicted hospital visits differed from the one used in our study. Second, we used morbidity data to model the need for health care instead of crude population counts. Third, provision of health care differs in the USA compared to Germany: Due to the mandatory health insurance in Germany, the influence of financial factors on the choice of a certain hospitals may not be as prominent as in the USA, where health insurance is not mandatory. Therefore, transferability of our results on an international level maybe dependent on the health care system in place. Regarding our approach to model the need for health care, due to possible under-, over- and misdiagnose, the modelled need may differ from the actual need. In addition, areas that lack adequate access to health care are potentially underdiagnosed due to the lack of access. Therefore, this bias could artificially increase correlations. However, our approach is widely used in health care research and represents a reasonable proxy for the need for health care [9, 12, 13]. Regarding our approach to measure accuracy in % of allowed error, it has to be noted that this approach focuses on areas with least error and ignores those with most errors, which limits its implications. Furthermore, uncertainties remain when area-based measures (such as spatial accessibility) are used to explain individual behaviors (choosing a certain hospital). These uncertainties are represented by the ‘modifiable areal unit problem’ (MAUP) and the ‘uncertain geographic context problem’ (UGCoP). In this context, it has been shown that scale effects of MAUP and UGCoP were minimal when area-level influence of healthcare accessibility on healthcare satisfaction are analyzed [43].

Beside the three FCA methods examined in this study (iFCA, M2SFCA and E2SFCA), there are other variations of FCA methods described in the literature [10, 20, 22, 44]. However, there is no consensus on which FCA method to use as the ‘gold standard’: all FCA methods mainly differ in their conceptualization of distance decay, which models the travel behavior of patients. The distance decay functions used in this study are furthermore only a small subset of possible distance functions used in the current literature [33]. Even if similar functions are used, the specific shape of the function is determined by its parameter settings. Therefore, the variety of functions and parameters makes comparisons difficult. A study has shown that sigmoidal functions are more likely suitable for densely populated areas whereas decline functions are more suitable for thinly populated areas [45]. However, choosing distance decay often lacks real world validation, mostly due to a lack of data [10, 33]. Hence, assumptions about the distance decay are often inevitable. The same is true for the catchment area. It has been shown that the choice of catchment area has a relevant impact on accessibility measures especially in urban areas [46]. In our study, we set the maximum catchment area to 120 min, which is in line with current literature [27]. However, smaller catchment areas (such as 90 min) have also been used [20]. By testing FCA methods using different decay functions, different parameter setting within each decay function, and different catchment areas settings, the understanding and performance of FCA methods to predict hospital visits could increase. Therefore, this issue should be evaluated in future studies.

Another issue with FCA methods is their static property as they account for population data and the location of health care services only at a specific time. However, population counts vary depending on daytime and the day of the week. These varying population counts are mainly due to commuting, leading to higher population counts in urban areas in contrast to residential counts [47]. Such commuting effects have not been accounted for in our study since we used residential morbidity data. In regard to both morbidity and population data, accounting for mobility patterns could potentially increase the accuracy of FCA measures [47]. We estimated disease burden based on ICD10-counts and population distribution using a disaggregation method. Such disaggregation methods are commonly applied in the absence of geocoded microdata [24]. However, the smaller the municipality (in terms of population size), the greater the uncertainty regarding the actual distribution of the disease burden. Therefore, the estimated distribution of the disease burden within a municipality may differ from the actual distribution especially in small municipalities.

The different approaches used among the FCA methods to predict hospital visits need further evaluation, especially in the context of their original application of measuring spatial accessibility. The accessibility index itself represents—in simplified terms—a ratio of hospital beds to hospital visits for each hospital (partial accessibility index) [10, 20, 48]. After computing the accessibility index, the predicted number of hospitals can be determined within the M2SFCA and the E2SFCA method. This would imply that for the first step (computing accessibility index), the ratio of hospital beds to hospital visits was based on an unrealistic calculation of hospital visits. However, this issue and its implication for the application of FCA methods in the prediction of hospital visits are not well studied yet. Thus, future research should focus not only on finding an appropriate measure of hospital attractiveness but also on the FCA methods used including their parameter settings.

## Conclusion

The potential benefit of the FCA methods, as shown in our study to predict hospital visits, could have a major impact on future health care planning. Knowing how many hospital visits should be expected if hospitals are closed, opened, or consolidated is crucial for health care planning. This is especially important since there is an emerging trend to consolidate hospitals in developed countries in order to use synergetic effects. In general, we were able to demonstrate the impact of FCA measures regarding the prediction of hospital visits especially in non-emergency settings. In addition, their superiority over commonly used methods such as the closest provider approach was shown. However, using hospital beds as the measure of hospital attractiveness resulted in low accuracy. More reliable measures of hospital attractiveness have to be used within the proposed methods to return more accurate results. Still, this study strengthens the possibilities of FCA methods in health care planning beyond their original application in measuring spatial accessibility.

## Data Availability

The datasets used and/or analysed during the current study are available from the corresponding author on reasonable request.

## Abbreviations

AI: Accessibility Index
FCA: Floating catchment area
M2SFCA: Modified 2 step floating catchment area
E2SFCA: Enhanced 2 step floating catchment area
iFCA: Integrated floating catchment area
ICD10: International statistical classification of diseases and related health problems
GDP: Gross domestic product
USA: United States of America
